# Implementation of a beating heart system for training in off-pump and minimally invasive coronary artery bypass

**DOI:** 10.1186/s12893-020-01023-z

**Published:** 2021-01-06

**Authors:** Shota Yasuda, Jef Van den Eynde, Katrien Vandendriessche, Munetaka Masuda, Bart Meyns, Wouter Oosterlinck

**Affiliations:** 1 Department of Cardiovascular Diseases, Research Unit of Cardiac Surgery, University Hospitals Leuven, Herestraat 49, 3000 Leuven, Belgium; 2 Department of Surgery, Yokohama City University, 3-9 Fukuura, Kanazawa-ku, Yokohama, Kanagawa 2360004 Japan

**Keywords:** Simulation training, Program evaluation, Coronary artery bypass, Anastomosis

## Abstract

**Background:**

Several training devices have been developed to train anastomotic skills in off-pump coronary artery bypass grafting (OPCAB). However, assessment of trainees’ improvement remains challenging. The goal of this study was to develop a new practical scoring chart and investigate its reliability and utility for anastomotic skills in OPCAB and minimally invasive direct coronary artery bypass (MIDCAB).

**Methods:**

A training device was used, which included a beating heart model installed in a dedicated box. A soft plastic tube was used as the left anterior descending artery, and a porcine ureter was used as the left internal mammary artery. Five cardiac surgery fellows (Fellows, > 5 year of surgical experience) and five residents or medical students (Residents, ≤ 5 year of surgical experience) were enrolled for this study. Before and after training, skills were evaluated using a scoring chart that took into account anastomotic time, leakage, shape, flow measurement, and self-estimation.

**Results:**

Mean total score of all trainees was 15.4 ± 4.0 at pre-training and 18.5 ± 2.4 at post-training (P = 0.05). Before training, there was a significant difference in the total score between Fellows and Residents (18.6 ± 2.2 vs 12.2 ± 2.4 points, P = 0.002), which disappeared after training (19.4 ± 2.5 vs 17.6 ± 2.2 points, P = 0.262). Residents benefitted from training with improvements in their time, total score, score for time, score for flow and subtraction score; however, these effects were not seen in Fellows. The most evident training effect was improvement of self-estimation, which was also seen in Fellows.

**Conclusions:**

Residents were most likely to derive benefit from these training models with regard to both efficiency and quality. Training models seem to have an important role in making surgeons feel more comfortable with the procedure.

## Background

Off-pump coronary artery bypass grafting (OPCAB) is a strategy for myocardial revascularization on a beating heart without the use of cardiopulmonary bypass. In experienced centers, OPCAB is associated with lower incidence of cerebral infarction, as well as lower need for hemodialysis and blood transfusion [1–3]. On the other hand, the technique is more challenging, and the learning curve for surgical trainees to perform an excellent anastomosis on a moving target should not be underestimated. As a result, centers with less experience tend to reduce the amount of anastomoses in order to simplify a complex operation. However, incomplete revascularization is associated with higher adverse cardiac event rates [4]. For this and other practical reasons, many hospitals prefer on-pump CABG, and the proportions of use between these two options have remained largely unchanged over the years [5]

Adequate technical skills training remains an essential part in the education of surgeons. Several training devices have been developed to train anastomotic skills on a beating heart model in a safe environment [6, 7]. However, few of these systems have been evaluated systematically in various settings. There is a lack of simulations for minimally invasive coronary artery bypass (MIDCAB), which is performed through smaller incisions rather than a full sternotomy [8]. Furthermore, without an appropriate scoring chart, it is difficult to evaluate to what extent these simulations contribute to the learning process of trainees.

Further optimization of the available beating heart simulations might provide useful tools to train young surgeons and keep track of the evolution of their skills. The goal of this study was therefore to develop a new practical scoring chart and investigate its reliability and utility for anastomotic skills in OPCAB and MIDCAB.

## Methods

This was a prospective, interventional cohort study of simulation as a training tool for acquisition of surgical skills. This study conforms to the ethical guidelines of the 1975 Declaration of Helsinki as reflected in a priori approval by the Medical Ethical Committee and Institutional Review Board (OG032) of the University Hospitals of KU Leuven (reference number ML10659). Training and testing took place at the Department of Cardiac Surgery of the University Hospitals of Leuven.

### Study subjects

Five fellows with more than five years of experience after graduating medical school (Fellows) and five residents or medical students with five or less years of experience (Residents) were enrolled for this study. All fellows had some experience with anastomosis techniques for coronary arteries, whereas none of the residents had such experience.

### Study devices

**Fig. 1:**
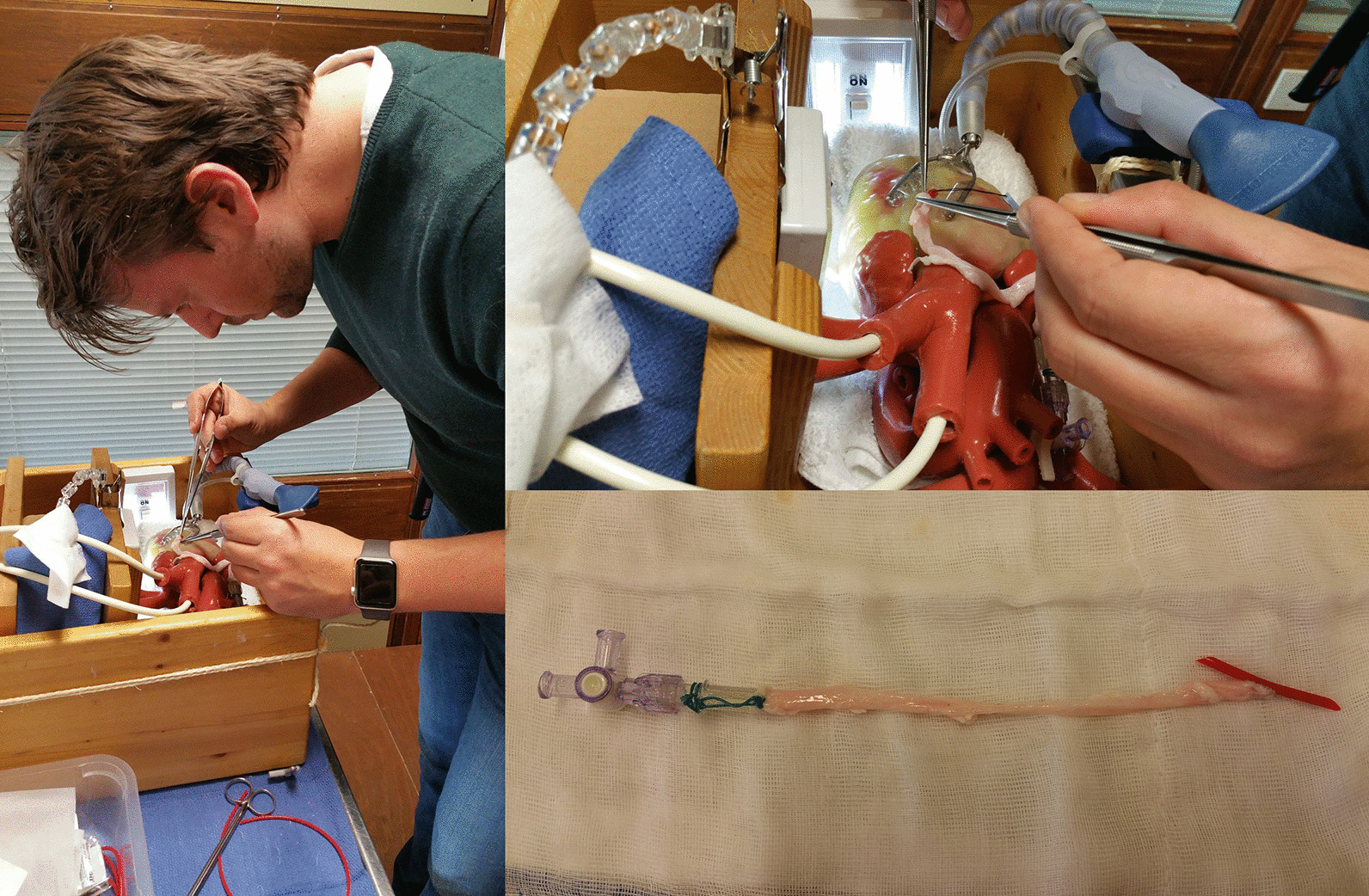
(Left panel) Trainee performing an anastomosis. (Right upper panel) Anastomosis using a pig ureter and a soft plastic tube as the left internal mammary artery to the left anterior descending artery. (Right lower panel) The pig ureter graft after completion of anastomosis

We used a beating heart model (CABG HEARTS#1259; The Chamberlain group, Great Barrington, MA, USA) and installed it in a dedicated box (Fig. 1) to mimic the spatial limitations in OPCAB. Beating of the heart was created by a compressor that compressed the air and adjusted the pressure in the heart, and the frequency of the beat was regulated by a dedicated regulator. A hollow soft plastic vessel loop (Ref. 18-474100-001, 1.5 mm × 3 mm × 1 m, Nootens H.Ets, Schaerbeek, Belgium) was used as the target vessel and was inserted in the gutter of the beating heart model (Fig. 1). The vessel loop was expanded and softened by scraping it with emery-paper. Pig ureters were harvested and preserved in a freezer at − 80 degree Celsius; they were defrosted before practice and used as the grafts. An Octopus heart stabilizer (Medtronic Plc, Dublin, Ireland) was installed on the beating heart. Each trainee used the same graft in order to ensure the same graft conditions.

### Training design

Trainees performed anastomoses from the left internal mammary artery to the left anterior descending artery with 8-0 polypropylene sutures and 1.75 mm shunt tubes (Fig. 1). The standard heart rate was set at 60 bpm. Following demonstration of the anastomosis technique by an expert (SY), trainees self-practiced the anastomosis independently at least three times. Before and after training, anastomotic skills were examined using a scoring chart (Table 1). All examinations were supervised and scored by SY.

**Table 1:** Scoring chart

	Time (min)	Leakage	Shape	Flow (ml/min)	Self-estimation
5 Points	< 8	None	Cobra head	> 200	Easy
4 Points	8–12	Oozing		151–200	Relatively easy
3 Points	12–16	1 Point leakage	Stenosis	101–150	Moderate
2 Points	16–20	2 Points leakage		51–100	Relatively difficult
1 Point	20 <	Dehiscence	Occlusion	< 50	Difficult
− 1 Point	Issues with the shunt tube				
− 2 Points	Issues with the needle or thread				

### Scoring chart

A new scoring chart was created to evaluate anastomotic skills based on clinically important parameters (Table 1). Items included: anastomotic time, leakage, shape, flow measurement, and self-estimation of the work. Each parameter was scored from 1 to 5 points (with 5 being full marks) and a total score was derived on a maximum of 25 points. Penalty points were given in case of issues with the shunt tube, or issues with the needle or thread (− 2 and − 1 points, respectively). Issues with the shunt tube were defined as unexpected shunt tube removal or transfixation of the thread through the shunt, whereas issues with the needle or thread were defined as unexpected cutting of the needle, or knot formation in the thread.

### Evaluation of anastomosis quality

The anastomotic site was evaluated after completion of the anastomosis. Saline was injected under 150 mmHg pressure while clamping both sides of the plastic tube, and verified for leakage from the anastomotic site. One leakage corresponded with a single jet from the suture line, two leakages corresponded with two jets from two points. If leakage was present, additional stitches were placed to allow for precise flow measurement subsequently. Simultaneously, the outer shape of the anastomotic site was inspected. A flow measurement device with a 4 mm flow probe (4 mm perivascular flow-probe PS series, Transonic Systems Inc. Ithaca, New York, USA) was used for the flow measurement after complete declamping of the proximal side of the plastic vessel loop (coronary artery), thus simulating a 100% coronary artery stenosis and ruling out any effects attributable to competitive flow. Finally, the graft was cut and the inside of the anastomosis was inspected for the presence of stenosis.

### Statistical analysis

Chart scores and time required for anastomosis were analyzed and compared between groups. Continuous variables were checked for normality and group differences were evaluated using t-test or Mann–Whitney U test accordingly. Results are expressed as the mean ± SD. A 2-way ANOVA was used to evaluate the difference between groups in relation to time (before and after training). Spearman’s rank correlation coefficient was used to evaluate the relationship between the change in time and change in score. All statistical analyses were performed using the SPSS software version 25 (SPSS Inc, Chicago, Illinois, USA). *P* values less than 0.05 were considered to indicate statistical significance.

## Results

### Effect of the training

**Fig. 2:**
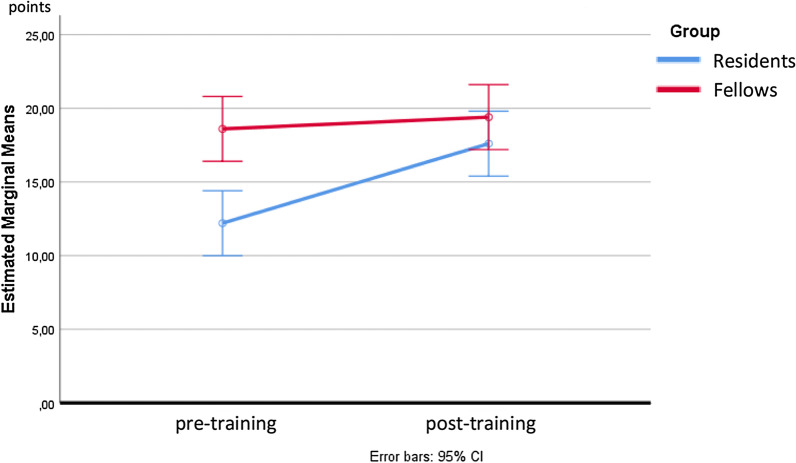
Estimated marginal means of total score. No significant difference was evident after the training

The mean total score of all trainees was 15.4 ± 4.0 at pre-training and 18.5 ± 2.4 at post-training (P = 0.05). The mean anastomotic time shortened from 21.1 ± 7.4 min before training to 17.1 ± 3.4 min after training (P = 0.144). Before training, there was a significant difference in the total score between the Fellows and Residents groups (18.6 ± 2.2 vs 12.2 ± 2.4 points, P = 0.002), which disappeared after training (19.4 ± 2.5 vs 17.6 ± 2.2 points, P = 0.262) (Fig. 2). After training, there was a significant improvement of total score, anastomotic time, flow, and self-estimation in the Residents group, with a significant reduction of penalty points (Tables 2 and 3). Even after excluding self-estimation from the scoring system (thereby only preserving the objectively measured factors), a clear benefit for residents was observed. Fellows showed improved self-estimation after training, but no improvements on other parameters.

**Table 2:** Results of the pre- and post-training assessment of residents’ and fellows’ performance

Item	Group	Performance		2-Way ANOVA		
		Pre-training	Post-training	Group effect	Training effect	Group × training interaction
Time (min)	Residents	26.8 ± 4.97	17.6 ± 2.51	F(1, 16) = 11,544	F(1, 16) = 4,805	F(1, 16) = 8.12
	Fellows	15.4 ± 3.97	16.6 ± 4.45	P = 0.004^a^	P = 0.044^a^	P = 0.012^a^
Score						
Total	Residents	12.2 ± 2.39	17.6 ± 2.19	F(1, 16) = 15,565	F(1, 16) = 8,898	F(1, 16) = 4,898
	Fellows	18.6 ± 2.19	19.4 ± 2.51	P = 0.001^a^	P = 0.009^a^	P = 0.042^a^
Time	Residents	1 ± 0	2 ± 0.71	F(1, 16) = 6.4	F(1, 16) = 1.6	F(1, 16) = 3.6
	Fellows	2.4 ± 0.89	2.2 ± 0.84	P = 0.022^a^	P = 0.224	P = 0.076
Leak	Residents	3.6 ± 1.52	3.4 ± 1.14	F(1, 16) = 0.036	F(1, 16) = 0.036	F(1, 16) = 0.321
	Fellows	3.2 ± 1.1	3.6 ± 0.89	P = 0.852	P = 0.852	P = 0.579
Shape	Residents	4.2 ± 1.1	4.6 ± 0.89	F(1, 16) = 3.6	F(1. 16) = 0,4	F(1. 16) = 0,4
	Fellows	5 ± 0	5 ± 0	P = 0.076	P = 0.536	P = 0.536
Flow	Residents	3.4 ± 1.67	4.8 ± 0.45	F(1, 16) = 4	F(1, 16) = 2.25	F(1, 16) = 4
	Fellows	5 ± 0	4.8 ± 0.45	P = 0.063	P = 0.153	P = 0.063
Self-estimation	Residents	1 ± 0	2.8 ± 0.45	F(1, 16) = 103,143	F(1, 16) = 48,286	F(1, 16) = 7,143
	Fellows	3.4 ± 0.55	4.2 ± 0.45	P = 0.000^a^	P = 0.000^a^	P = 0.017^a^
Penalty points	Residents	− 1 ± 1	0 ± 0	F(1, 16) = 0.095	F(1, 16) = 2,381	F(1, 16) = 2,381
	Fellows	− 0.4 ± 0.55	− 0.4 ± 0.89	P = 0.762	P = 0.142	P = 0.142
Total score excluding self-estimation Self sstimation	Residents	11.2 ± 2.39	14.8 ± 2.17	F(1, 16) = 5.29	F(1, 16) = 3,541	F(1, 16) = 3,541
	Fellows	15.2 ± 1.79	15.2 ± 2.17	P = 0.035^a^	P = 0.078	P = 0.078

Number of subjects: fellows = 5, residents = 5

*ANOVA* analysis of variance

^a^Statistically significant, P < 0.05

**Table 3:** Estimated effect of training for residents and fellows

Item	Group	Estimated marginal means		
		Mean difference	P-value	Partial η squared
Time (min)	Resident	− 9.2 ± 2.58	0.003^a^	0.44
	Fellow	1.2 ± 2.58	0.648	0.01
Score				
Total	Resident	5.4 ± 1.47	0.002^a^	0.46
	Fellow	0.8 ± 1.47	0.594	0.02
Time	Resident	1 ± 0.45	0.04^a^	0.24
	Fellow	− 0.2 ± 0.45	0.661	0.01
Leak	Resident	− 0.2 ± 0.75	0.793	0.00
	Fellow	0.4 ± 0.75	0.6	0.02
Shape	Resident	0.4 ± 0.45	0.384	0.05
	Fellow	0 ± 0.45	1	0.00
Flow	Resident	1.4 ± 0.57	0.025^a^	0.28
	Fellow	− 0.2 ± 0.57	0.728	0.01
Self-estimation	Resident	1.8 ± 0.27	0.000^a^	0.74
	Fellow	0.8 ± 0.27	0.008^a^	0.36
Penalty points	Resident	1 ± 0.46	0.044^a^	0.23
	Fellow	0 ± 0.46	1	0.00
Total score excluding self-estimation	Resident	3.6 ± 1.35	0.017^a^	0.31
	Fellow	0 ± 1.35	1	0.00

^a^Statistically significant, P < 0.05

### Correlation between change in time and change in score

**Fig. 3:**
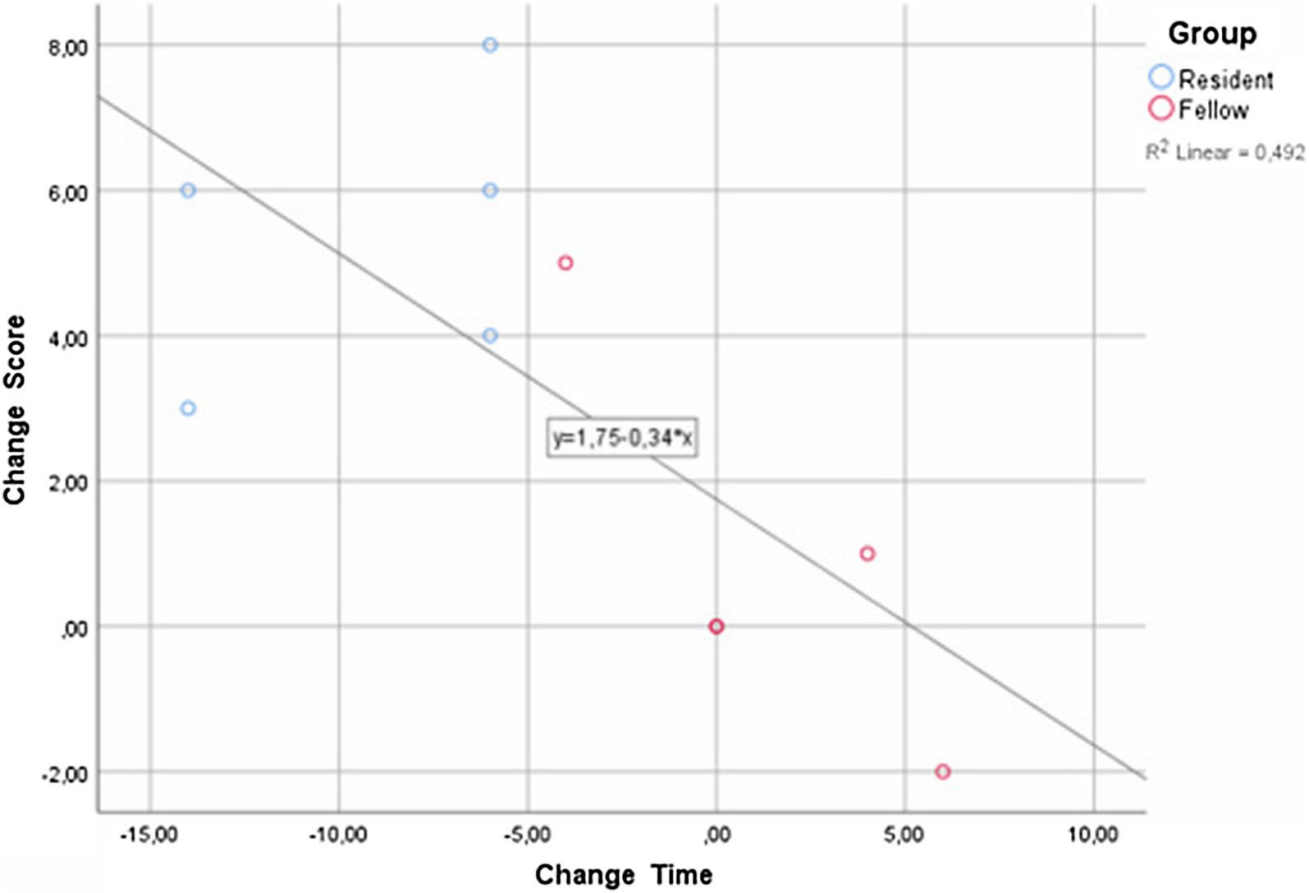
Correlation between change in time and change in score with scatter plot and regression equation

Some degree of correlation between the change in time and change in score was noted (score = 1.75–0.34*time, R^2^ = 0.492) (Fig. 3). Residents showed the most evident changes, whereas fellows did not show any marked improvement in their score or time after training.

## Discussion

Technical skills training remains an essential part of surgical education. Simulation models are a promising tool, especially in procedures as challenging as OPCAB and MIDCAB. In this study, we used a beating heart model and developed a new score chart in order to further optimize training and make the learning process more trackable.

A first finding of our study was the significant difference in total score between the Fellows and Residents groups before training, which disappeared after training. Indeed, all Fellows had had experience with anastomotic techniques for coronary revascularization in the past. This suggests that our scoring chart might give an accurate estimation of trainees’ initial skill level, and that every increase in their score might reflect improvement of skills. This allows supervisors to evaluate the level of knowledge when fellows or residents arrive at their department, which can facilitate individually tailored training. In addition, it allows monitoring of progress, which can be motivational for both trainees and supervisors.

In 2008, Fann et al. proposed a first performance rating score for coronary anastomosis on beating heart simulators [6]. The 8-item score was modified from the Objective Structured Assessment of Technical Skills (OSATS) described by Reznick et al. [9] and showed clear improvement on various technical aspects with training. However, the scoring system is complex and does not lend itself to quick periodic assessment. Furthermore, leakage, anastomosis shape, and intraoperative graft flow measurements are not accounted for in the score, although these represent major quality checks performed in real life surgery. In addition, trouble with the shunt tube and suture thread that can occur during surgery can lengthen the anastomotic time and reduce quality. Trainees’ ability to perform coronary anastomoses may therefore be more precisely represented by taking these frequent troubles into account when determining the score. The advantage of our scoring system is that it allows for a quick assessment, taking into account all factors that will determine bypass quality in real life.

In our study, residents were the most likely to derive benefit from these training models with regard to both efficiency (time, self-estimation) and quality (flow). The most evident training effect was improvement of self-estimation, which was also seen in fellows. This suggests that training models might have an important role in making surgeons feel more comfortable with the procedure, which is conducive to preparing them for more advanced surgical procedures in the operating theatre. Indeed, once a surgeon has gained self-confidence over one part of the procedure, less effort has to be invested in that part of the surgery and more energy will be available for other pats of the surgery which require attention. It is unclear why fellows’ skills did not markedly improve with this training in our study. The system might not have been challenging enough, given their previous experience with anastomosis techniques. As the fellows were used to advanced surgical instruments and the specific conditions of a clinical operating theatre, adjusting to the specific experimental set-up could have attenuated their true improvement with training. However, as fellows did not obtain the maximum score, additional training could allow further monitoring and improvement of their level to an expert level.

A limitation of our study is that some features of CABG have not been included in our model: consideration of graft design, graft harvesting, creation of an operating field, fixation of the coronary artery with a stabilizer, and dissection and opening of the coronary artery [10, 11]. All these techniques are mandatory for CABG in general and are not unique for OPCAB. However, given that the main difficulty of OPCAB compared to on-pump CABG lies in the need to operate on moving target vessels in a deep operating field, we are convinced that practicing an anastomosis within these specific conditions should receive major attention when training young surgeons to perform OPCAB. Furthermore, improved flows and bypass quality will benefit patient outcomes in both OPCAB and on-pump CABG. Another limitation is that we only tested performance of LIMA-LAD anastomoses. For further practice, our model could be expanded to include also the evaluation of the circumflex and right coronary artery. Finally, our study only evaluated the effect of practice on the short term, whereas training usually takes place over a much longer period. However, here we aimed to evaluate the short-term efficacy of our training model and to validate our scoring system. Our institution is currently enrolling a randomized controlled trial to study the long-term efficacy of our training model.

Several training kits for CABG have been introduced, yet the issue of continuous costs remains [8, 12]. In our training system, the only required investment is the beating heart system; all other material is mainly re-usable. The ureters of the pig are harvested as waste from other acute experiments, which avoids ethical issues as well as recurring additional costs.

The plastic tube is made of a relatively hard material which resembles atherosclerotic native vessels. It can be made softer by scraping the surface of the tube. Ureters of pigs have a tapered structure, where the distal side is slender and similar to an internal mammary artery and the proximal side is thick and like a saphenous vein graft. This combination of tubes and ureters allows for the replication of various situations for trainings.

**Fig. 4:**
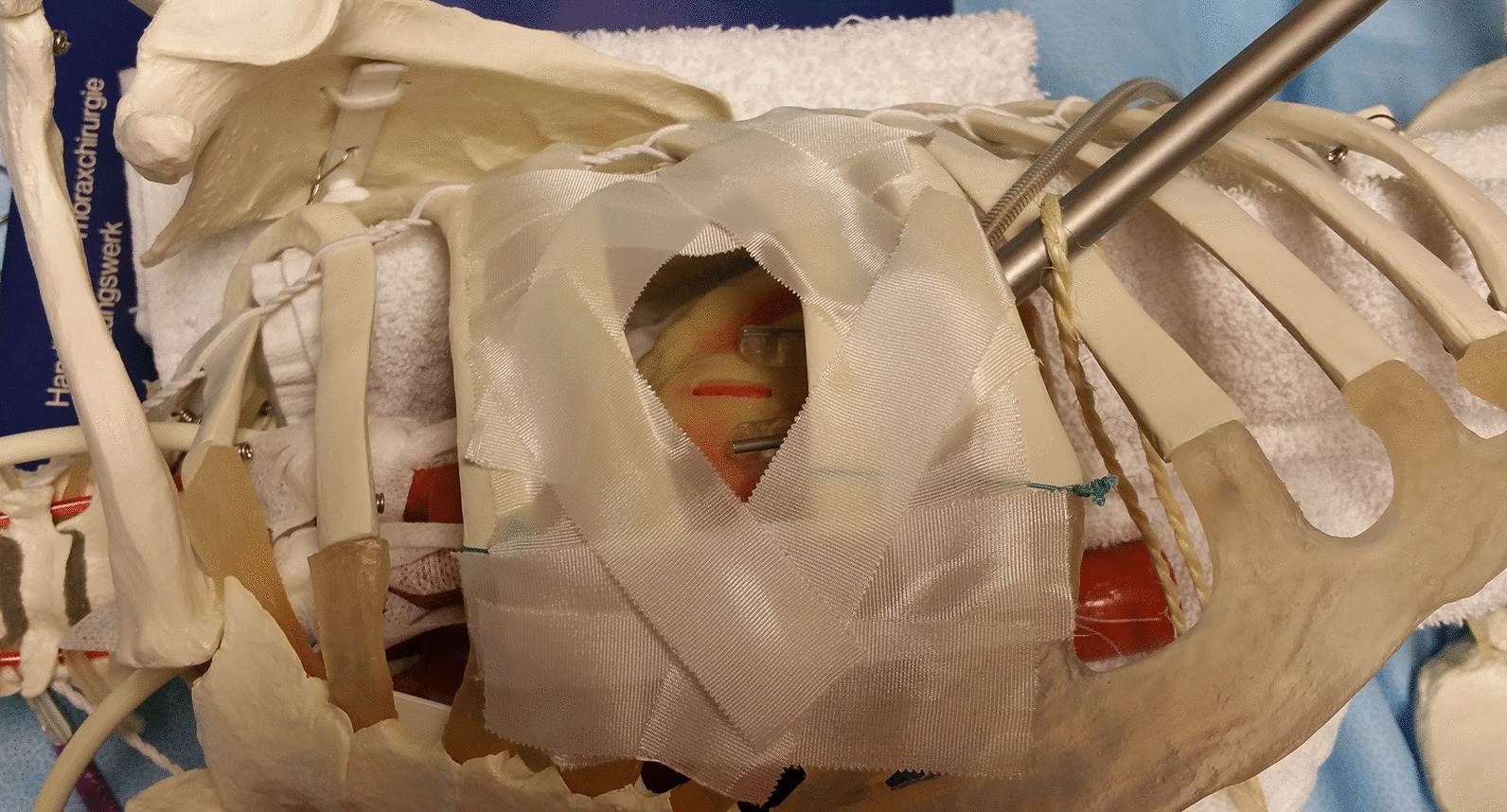
MIDCAB training model using skeleton body

Some reports maintained that on-the-job training for their fellows in cardiac surgery had a good result without any increased mortality [13, 14]. From an ethical perspective, however, this remains a difficult point, especially when the exact performance level of the trainee still needs to be determined. Time efficacy also requires that trainees are maximally prepared before they start working on living subjects. In addition, MIDCAB is receiving renewed focus with the advent of robotic surgery [15, 16] and the gained interest in hybrid approaches [17, 18]. As an example, a novel option for cases of triple vessels disease is the performance of LIMA-LAD anastomosis by robotic assisted MIDCAB followed by percutaneous coronary intervention (PCI) for the other two coronary vessels [19] Given the limited space through a thoracotomy, young surgeons who are eager to become proficient in performing MIDCAB must practice coronary anastomosis using appropriate training devices, to obtain enough experience and ease so that they can perform adequately under high pressure and ensure proper bypass quality. To this purpose, we have developed a setting to our simulation specifically for MIDCAB using a skeleton model in which the beating heart can be placed (Fig. 4). This advanced device may allow fellows to engage in more effective trainings than is available at present and to give them additional challenging training goals.

## Conclusions

In conclusion, this study showed that training on a beating heart model improved coronary anastomosis skills in residents and improved confidence about surgical performance in both residents and fellows. The training situation can be adapted to specifically represent features of OPCAB and MIDCAB. Furthermore, a new scoring chart was introduced, which might be useful for both quick initial assessment of new residents and tracking of their progress.

## Data Availability

The datasets used and/or analyzed during the current study are available from the corresponding author on reasonable request.

## Abbreviations

CABG: Coronary artery bypass grafting
LAD: Left anterior descendens
LIMA: Left internal mammary artery
MIDCAB: Minimally invasive direct coronary artery bypass grafting
OPCAB: Off-pump coronary artery bypass grafting
OSATS: Objective structured assessment of technical skills
PCI: Percutaneous coronary intervention
